# Right heart echocardiography findings in hypoxic pneumonia patients during the COVID-19 pandemic in a South African population

**DOI:** 10.1093/ehjimp/qyad030

**Published:** 2023-10-25

**Authors:** S A van Blydenstein, S Omar, B Jacobson, C N Menezes, R Meel

**Affiliations:** Division of Pulmonology, Faculty of Health Sciences, Chris Hani Baragwanath Academic Hospital, University of the Witwatersrand, Chris Hani Road, Johannesburg, 1864, South Africa; Division of Critical Care, Faculty of Health Sciences, Chris Hani Baragwanath Academic Hospital, University of the Witwatersrand, Chris Hani Road, Johannesburg, 1864, South Africa; Division of Haematology, National Health Laboratory Service, Faculty of Health Sciences, University of the Witwatersrand, Johannesburg, 2193, South Africa; Division of Infectious Diseases, Faculty of Health Sciences, Chris Hani Baragwanath Academic Hospital, University of the Witwatersrand, Chris Hani Road, Johannesburg, 1864, South Africa; Department of Internal Medicine, Faculty of Health Sciences, University of the Witwatersrand, Johannesburg, 2193, South Africa

**Keywords:** COVID-19, pneumonia, right heart strain, right ventricle, predicted mortality, right ventricular free wall strain

## Abstract

**Aims:**

The right ventricle is affected by Coronavirus disease 19 (COVID-19) via multiple mechanisms, which can result in right ventricular dysfunction (RVD). This study aimed to provide an assessment of right heart function using conventional echocardiography and advanced strain imaging, in patients with hypoxic pneumonia during the COVID-19 pandemic.

**Methods and results:**

This study was an observational, prospective, single-centre study, including adults with hypoxic pneumonia, in two groups: COVID-19 pneumonia; and non-COVID-19 pneumonia. Bedside echocardiography was performed according to a pre-specified protocol and all right heart measurements were done as per standard guidelines. Right ventricular free wall strain (RVFWS) was measured using Philips® QLAB 11.0 speckle tracking software. Descriptive and comparative statistics were used to analyse data. Spearman Rank Order Correlations were used to determine the correlation between right ventricular (RV) parameters and clinical parameters. Univariate and multivariate logistic regression analyses were performed to characterize the predictors of in-hospital mortality. We enrolled 48 patients with COVID-19 pneumonia and 24 with non-COVID-19 pneumonia. COVID-19 patients were significantly older with a higher frequency of hypertension and diabetes and a trend towards a lower severity of illness score. Mean RVFWS yielded the highest estimates for the prevalence of RVD (81%), with no difference between the two pneumonia groups. Median Tricuspid Annular Plane Systolic Excursion (TAPSE) and right ventricular systolic excursion velocity (RVS’) were not significantly different between COVID-19 (TAPSE 17.2 and RVS’ 12), and non-COVID-19 pneumonia (TAPSE 17.8 and RVS’ 12.1) with *P* values of 0.29 and 0.86, respectively. Non-COVID-19 pneumonia patients with moderate to severe hypoxaemia (PF < 150) were at greater risk of an elevated RV Systolic Pressure >30 mmHg respiratory rate = 3.25 (CI 1.35–7.82) on admission. Troponin levels discriminated between COVID-19 survivors (6 ng/L) and non-survivors (13 ng/L), *P* = 0.04. The mortality rate for COVID-19 was high (27%) compared to non-COVID-19 pneumonia (12%).

**Conclusion:**

Patients with COVID-19 pneumonia had a similar admission prevalence of RVD when compared to patients with non-COVID-19 pneumonia. Despite preserved traditional parameters of RV systolic function, RVFWS was diminished in both groups, and we propose that RVFWS serves as an important marker of the subclinical disease of RV.

## Graphical Abstract

**Graphical abstract:**
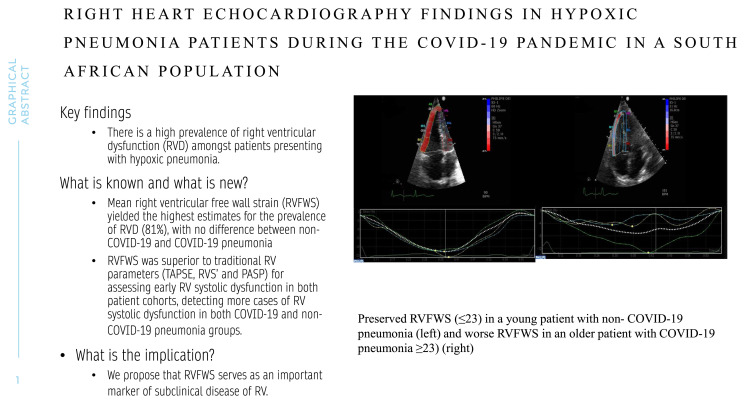

## Introduction

The right ventricle is affected by Coronavirus disease 19 (COVID-19) by multiple mechanisms, including cardiac and systemic inflammation, volume status, increased sympathetic tone, direct cardiac involvement by severe acute respiratory syndrome coronavirus 2 (SARS-Co-V-2), and thrombosis (including micro-thrombosis and macro-thrombosis). This alters the ventilation-perfusion matching resulting in ventilation/perfusion mismatching, hypoxia from shunting and atelectasis, and hypoxic pulmonary vasoconstriction increasing right ventricular (RV) afterload.^1^ This can cause acute cor pulmonale, cardiogenic shock, thrombotic events and complications, acute coronary syndromes, myocardial injury, and arrhythmias.^2^ Findings on transthoracic echocardiography of hospitalized COVID-19 patients relevant to the right heart include increased pulmonary arterial pressures (PAP);^2,3^ RV dilatation;^4^ and RV dysfunction (RVD).^3,5^ Mortality in severe COVID-19 pneumonia with a diagnosis of RVD is increased compared to those without RVD.^4,6^

There has been a high incidence of venous thromboembolism described in COVID-19,^7,8^ often despite thromboprophylaxis administration,^9^ with microthrombi found in small lung arteries,^10^ and a raised d-dimer in non-survivors compared to survivors.^2^ There is increased arterial thrombosis manifesting as an increased rate of acute myocardial infarction.^11^ The vasculopathy of the lung likely manifests in ventilation/perfusion mismatching and contributes to the hypoxaemia seen in COVID-19 pneumonia.^12^

The RVD may be caused by COVID-19 sequelae, including hypoxic pulmonary vasoconstriction, which characterizes COVID-19-related pneumonia and acute respiratory distress syndrome (ARDS).^13^ Progressive consolidation and atelectasis cause further physical distortion of the pulmonary vessels,^14^ and thromboembolism, and the hypercoagulopathy seen in COVID-19 add additional strain and increase pulmonary arterial systolic pressure (PASP). Furthermore, should the patient receive positive end-expiratory pressure and/or driving pressure, the afterload to the RV is increased. These mechanisms all contribute to the increased RV strain. Additionally, there is evidence to show that there is direct myocardial involvement in COVID-19.^10,15^

Traditionally RV function has been characterized using Tricuspid Annular Plane Systolic Excursion (TAPSE), the RV fractional area change, and the RV systolic excursion velocity (RVS’), and is a predictive factor of mortality in COVID-19,^16^ however, RV free wall strain (RVFWS) is a better predictor of mortality than TAPSE and RVS’.^16^ The current non-invasive gold standard used to describe RV function is cardiac MRI,^17^ however, it is expensive, not readily available, and not appropriate for use during a pandemic.

There is a paucity of data regarding RV function in COVID-19 pneumonia from Africa and therefore our main aim was to provide a detailed assessment of RV function amongst patients admitted with hypoxic pneumonia using conventional echocardiography and advanced strain imaging and to describe the differences between COVID-19 and non-COVID-19 hypoxic pneumonia with regard to RV involvement.

## Methods

### Study design and site

This was a prospective, observational, cohort study of hypoxic pneumonia including COVID-19 pneumonia, and non-COVID-19 pneumonia in adult patients at Chris Hani Baragwanath Academic Hospital (CHBAH), South Africa.

### Study population, definitions, inclusion, and exclusion criteria

We screened all consecutive adult patients during working hours on weekdays, who were persons under investigation for SARS-CoV-2 virus, and were admitted between 20 October 2020 and 11 March 2021. Patients were included if they had hypoxic physician-diagnosed or chest x-ray (CXR)-diagnosed pneumonia and met the criteria for severe disease or critical illness. Severe disease was defined as oxygen saturation ≤92% with a respiratory rate ≥30 and therefore requiring supplemental oxygen support without the need for invasive or non-invasive ventilation.^18^ Critical illness was defined as hypoxaemia and the need for additional ventilatory support, in the form of non-invasive ventilation.^18^ The study investigation was performed within 48 h from admission and before the initiation of invasive mechanical ventilation. There was no serial imaging, outcome was determined as either survival (discharge from hospital) or death and was assessed retrospectively, with no intervening imaging or clinical follow-up during the admission pertinent to the study. We excluded patients if they had *mycobacterium tuberculosis* and *Pneumocystis jiroveci pneumonia*, were pregnant, had a known chronic lung disease, chronic cardiac disease, specifically ischaemic heart disease, cardiomyopathy, had organic valvular heart disease, either known or incidental, or had a history of pulmonary or cardiac surgery, and if the imaging was poor quality. This study focussed on right heart echocardiographic findings and excluded all patients with a left ventricular ejection fraction (LVEF) < 50%, in an attempt to exclude right heart abnormalities due to left heart systolic dysfunction.

Patient demographic, clinical, laboratory, and hospital survival data were extracted from clinical notes. The following scores were calculated from admission data: simplified acute physiology score 2 (SAPS2),^19^ and Sequential Organ Failure Assessment (SOFA) scores,^20^ We further classified patients using the Horowitz Index for Lung Function: ratio of arterial oxygen partial pressure (PaO_2_ in mmHg) to fractional inspired oxygen (FiO_2_) expressed as a fraction (P/F) ratio of below 150 or ≥ 150.

#### Two- dimensional echocardiography

Transthoracic echocardiography was performed on all patients in the left lateral or supine position using an S5-1 transducer on a Philips^®^ portable CX 50 system (Amsterdam, the Netherlands). The images were obtained according to a standardized protocol. The data were transferred and analysed offline using the Philips^®^ Xcelera workstation. In addition to LVEF, the following RV systolic functional parameters were measured: A pulse-wave tissue doppler imaging (TDI) was performed in the apical four-chamber view using a sample volume size (6 mm) adequate to cover the basal RV longitudinal excursion, and we measured time from beginning of isovolumetric contraction to the peak of the S wave in TDI (time to peak) to non-invasively estimate the mean pulmonary pressure as a surrogate for pulmonary hypertension as compared to right heart catheterization.^21^ The cut-off time used to define pulmonary hypertension was 127 ms.^21^ RV pulse-wave Doppler was performed to assess the RV E/A ratio and E/E′ ratio, as a surrogate of RV diastolic function. All measurements relating to the RV were performed by an experienced Cardiologist based on the ASE guidelines and using the guidelines on the RV for cut-off of RVD^22^ and the values described in the specific population.^23^ RVFWS cut-off of −23 was used and has been feasible and reproducible in our unit with intra-observer and inter-observer variability coefficients of 7% and 7.6%, respectively.^24^

#### Right ventricle strain analysis

RVFWS was derived from a modified A4C RV view. Once three points, namely the RV apex, medial, and lateral tricuspid annulus, were defined, the software automatically traced the endocardial and epicardial border. Philips^®^ QLAB version 11.0 software allowed offline semi-automated analysis of speckle-based strain. This results in the division of the RV into six standard segments in the A4C view. The region of interest, once created, can be manually adjusted as needed to allow for adequate speckle tracking. RVFWS is less impacted by cardiac motion, the left ventricle, and the volume status,^25,26^ and gives both regional and average assessments. RVFWS has previously been shown to be more sensitive at diagnosing RVD in some conditions, than TAPSE and Doppler tissue imaging.^27^ The RVFWS was obtained by averaging three lateral segments (the basal, mid, and apical RV wall). A cut-off of −23% was used, with more negative values indicating better myocardial mechanics.^28^ The interventricular septum was excluded from the analysis. The longitudinal ɛ curves for each segment and a mean curve of all segments were generated by the software. These curves were used to derive peak negative RVFWS.

### Outcome measures

The primary outcome was to describe the prevalence of RV dysfunction. The secondary outcomes included a description of RV dimensions, RV function, the relationship between RV function, and troponin, and D-Dimer, to describe the relationship between RVFWS and oxygenation, and to explore the predictors of mortality.

### Data collection

Study data were collected and managed using REDCap^®^ electronic data capture tools hosted at the University of Witwatersrand (Research Electronic Data Capture).^29,30^

### Statistical analysis and sample size

Statistical analyses were performed using Statistica^®^ version 13.3 (TIBCO Software Inc., USA). Continuous variables were expressed as median [interquartile range (IQR)], and proportions/percentages were used for categorical variables. Continuous data were compared using the Mann–Whitney *U* test while proportions were compared using the chi-square test. A *P*-value < 0.05 was considered statistically significant. Spearman Rank Order Correlations were used to determine the correlation between RV parameters and clinical parameters. Univariate and multivariate analyses using logistic regression analysis were performed to characterize the predictors of in-hospital mortality using biochemical and echocardiographic factors. A convenience sample was employed.

### Ethics considerations

Approval was received from the University Human Research Ethics Committee (Medical), M200728, and is registered on the National Health Research Database, South Africa, GP_202008_140. Written informed consent from the patient or surrogate was obtained as per local ethics committee guidelines.

## Results

### Patient characteristics

**Figure 1:**
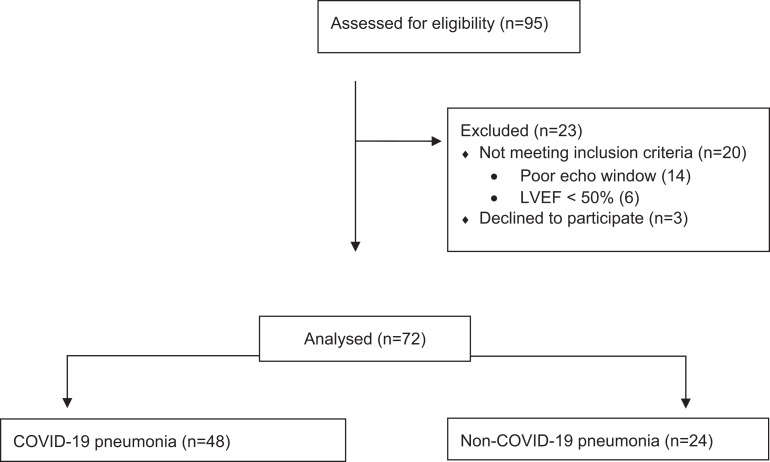
Study participation flow diagram.

We enrolled 72 hypoxic pneumonia patients between 20 October 2020 and 11 March 2021. We enrolled 48 patients with COVID-19 pneumonia (COVID-19) and 24 with non-COVID-19 pneumonia. *Figure 1* describes the participant selection process. The clinical characteristics of the cohort are shown in *Table 1*. Baseline arterial blood gas and biomarkers are provided in *Table 2*. Compared to the COVID-19 cohort, the non-COVID-19 cohort had significantly higher d-dimers. There was a weak correlation between troponin and RV diastolic dysfunction [RV E/A 0.26 (*P* < 0.05) and RV E/E′ 0.27 (*P* < 0.05)].

**Table 1:** Patient profile

Variable	Total	Non-COVID-19 pneumonia	COVID-19 pneumonia	*P*-value
	Mean (SD)/median [IQR], *n* = 72	Mean (SD)/median [IQR], *n* = 24	Mean (SD)/Median [IQR], *n* = 48	
Age (years)	49 (13.6)	42.9 (11.6)	52 (42–62.5)	0.006^a^
Female, *n* (%)	40/72 (56)	12/24 (50)	28/48 (58)	0.5
Co-morbidities (%)				
HIV	25/72 (35)	13/24 (54)	12/48 (25)	0.01^a^
Hypertension	30/72 (42)	4/24 (17)	26/48 (54)	0.002^a^
Diabetes	11/70 (16)	2/24 (8)	9/48 (19)	0.04^a^
Renal dysfunction	2/72 (2.8)	1/24 (4.2)	1/48 (2.1)	0.61
Other	20/72 (28)	6/24 (25)	14/48 (29)	0.71
Smoker	15/72 (21)	7/24 (29)	8/48 (17)	0.21
SAPS II	21 [14–34]	29 [17–36.5]	18 [13–31.5]	0.15
SOFA	2 [2–4]	2.5 [2–4]	2 [2–4]	0.36
Lactate mmol/L	1.8 [1.2–2.4]	2.2 [1.7–3.2]	1.6 [1.1–2.4]	0.03^a^

SD, standard deviation; *n*, number; HIV, human immunodeficiency virus; SAPS II, simplified acute physiology score 2; SOFA, Sequential Organ Failure Assessment.

^a^Statistically significant.

**Table 2:** Admission blood gas variables, biomarkers, and total lung ultrasound score

Variable	All		Non-COVID-19 pneumonia			COVID-19 pneumonia	*P*-value
	*n*	Mean (SD)/median [IQR]	*n*	Mean (SD)/median [IQR]	*n*	Mean (SD)/median [IQR]	
PaO_2_ mmHg	72	45 [31–62]	24	43 [29–57]	48	46 [32–63]	0.47
FiO_2_	72	0.33 [0.21–0.6]	24	0.21 [0.21–0.5]	48	0.4 [0.21–0.60]	0.14
P/F ratio	72	132 [93–192]	24	143 [96–218]	48	126 [81–183]	0.62
pH	72	7.43 [7.40–7.46]	24	7.42 [7.38–7.45]	48	7.44 [7.40–7.46]	0.38
PaCO_2_ mmHg	60	37 (7.4)	24	36 (9.4)	45	37 (8.5)	0.83
BE meq/L	70	0.3 [−3.0–3.9]	24	−2.7 [−4.1–3.9]	46	1.3 [−1.5–4.0]	0.13
CRP mg/L	72	128 [60.5–211]	24	106 [63–225]	48	132.5 [56–192]	0.92
d-dimer mg/L	66	1.15 [0.38–3.56]	21	2.08 [1.08–3.84]	45	0.82 [0.35–3.18]	0.02^a^
Troponin ng/L	62	8 [5–20]	16	9 [5–19]	46	7 [5–21]	0.65
Total LUS	72	8 [4–12]	24	7.5 [4.4–12.5]	48	8 [4–11.5]	0.56

[IQR], interquartile range with median; (SD), standard deviation with mean; CRP, C reactive protein; PaO_2_, partial pressure of oxygen; FiO_2_, fraction of inspired oxygen; P/F, Horowitz index for Lung Function (P/F ratio); PaCO_2_, partial pressure of carbon dioxide; BE, base excess.

^a^Statistically significant.

### Primary outcome

**Figure 2:**
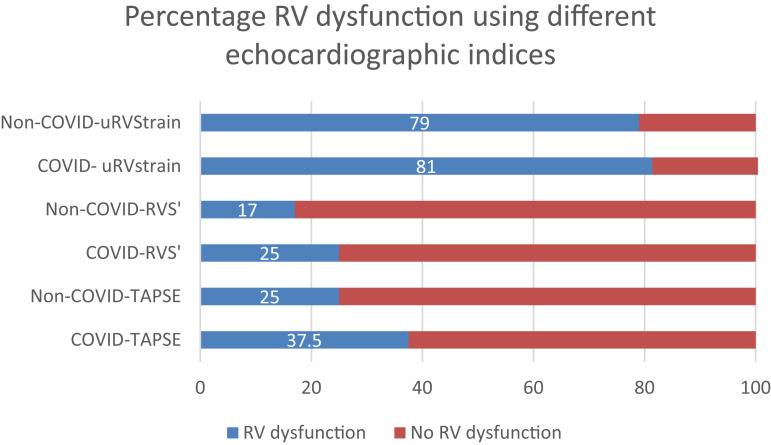
Prevalence (%) of RV dysfunction according to traditional echocardiographic parameters (RVS’ and TAPSE) and RVFWS amongst COVID-19 and non-COVID-19 patients at admission.

**Figure 3:**
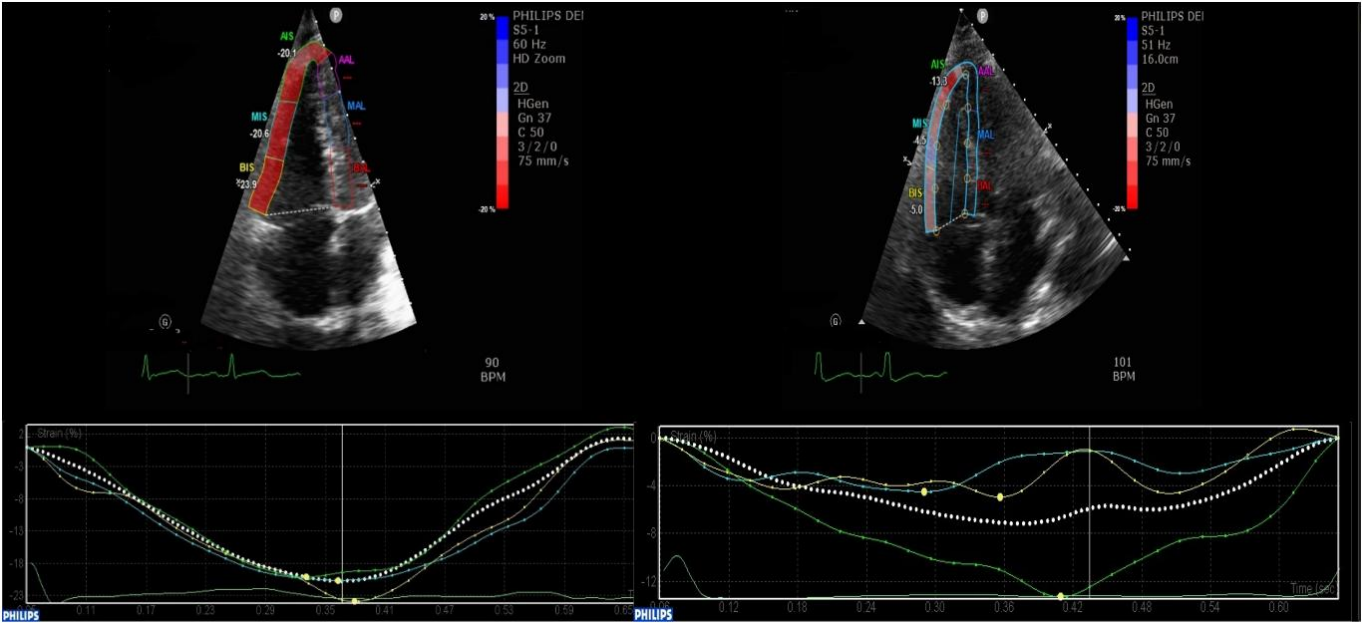
Preserved RV-free wall peak systolic strain (<–23) in a young patient with non-COVID-19 pneumonia (left) and worse RV-free wall peak systolic strain in an older patient with COVID-19 pneumonia (>–23) (right).

The highest overall percentage prevalence of RVD was obtained using RVFWS, 81% [confidence interval (CI) 76–85%, *n* = 67]. *Figure 2* demonstrates the percentage prevalences of RVD using different echocardiographic methods among COVID-19 and non-COVID-19 pneumonia participants. *Figure 3* demonstrates the measurement of RVFWS in two patients from the cohort.

### Secondary outcomes

Right ventricular wall thickness and inferior vena cava (IVC) diameter were both significantly higher in the non-COVID-19 pneumonia group, shown in as *Table 3*. Despite a statistically greater E/A ratio in the non-COVID-19 compared to the COVID-19 pneumonia group, both median values were within the reference range. There was a statistically significant, weak negative correlation between Troponin T and RV E/A ratio (*r* = −0,26, *P* < 0.05), a weak positive correlation with D-Dimer [*r* = 0.28, *P* < 0.05)]. RVS’ was weakly negatively correlated with total lung ultrasound score (LUS) (*r* = −0.25, *P* < 0.05). Finally, time to peak inspiratory pressure was statistically significantly negatively correlated to total LUS (*r* = −0.39, *P* < 0.05).

**Table 3:** Echocardiography findings

Variables	*n*	All	*n*	COVID-19 pneumonia	*n*	Non-COVID-19 pnuemonia	*P* value
*Right ventricular dimensions*							
RV wall thickness (cm)	69	0.60 [0.56–0.74]	45	0.60 [0.54–0.67]	24	0.72 [0.58–0.81]	0.04^a^
RV base diameter (mm)	69	31.4 [27.4–36.2]	45	31.6 [27.1–36.8]	24	30.3 [27.5–35.2]	0.70
RV longitudinal diameter (mm)	69	60.5 [53.4–67.5]	45	58.7 [51.7–67.2]	24	65.5 [56.8–68.5]	0.08
Tricuspid annular size	70	28.97 (±5.02)	47	28.54 (±4.9)	23	29.83 (±5.3)	0.32
IVC diameter (mm)	69	11.2 [9–13.8]	45	10.2 [8.6–12.1]	24	13.3 [9–15.3]	0.03^a^
RA major dimension (mm)	70	41.8 (±7.1)	46	42.3 (±7.1)	24	40.8 (±7.1)	0.42
RA minor dimension (mm)	69	35.4 (±7.2)	46	34.4 (±6.6)	23	37.4 (±8.1)	0.10
*Right ventricular function indices*							
Time from isovolumetric contraction to the peak of the S wave (TTP) (ms)	71	99 [83–117]	47	99 [83–112]	24	93.5 [83–144]	0.62
TAPSE (mm)	72	17.4 [15.2–21.8]	48	17.2 [14.9–21.6]	24	17.8 [16.2–22.2]	0.29
RVSP (mmHg)	53	10 [10–23]	32	10 [10–17.7]	21	15 [7–25]	0.75
RVS’ (cm/s)	72	12 [10–14.5]	48	12 [9.8–14.9]	24	12.1 [10.2–13.0]	0.86
E velocity cm/s	72	54.17 (±16.44)	48	53.29 (±15.89)	24	55.93 (±17.71)	0.52
A velocity cm/s	71	61.09 (±18.83)	48	63.98 (±17.91)	23	55.05 (±19.65)	0.06
E/A	71	0.84 [0.72–1.14]	48	0.83 [0.69–0.99]	23	1.12 [0.76–1.54]	0.02^a^
*E*′ velocity cm/s	70	10.42 (±2.96)	46	9.70 (±2.36)	24	11.82 (3.51)	0.003^a^
*A*′ velocity cm/s	71	14.2 [11.5–16.5]	47	13.8 [11.5–15.8]	24	14.7 [11.4–17.6]	0.69
*E/E*′	70	5.32 [3.99–6.63]	46	5.44 [4.29–7.04]	24	4.56 [3.61–6.34]	0.21
RV free wall strain (%)	68	−14.4 [−18.9 to −9.7]	44	−14.6 [−18.8 to −10.5]	24	−12.9 [−19.3 to −9.1]	0.97

^a^Statistically significant; [IQR], interquartile range with median; (SD), standard deviation with mean, interquartile range; ms, milliseconds; mm, millimetres; mmHg, millimetre mercury; *n*, number, TAPSE Tricuspid Annular Plane Systolic Excursion, RVSP, right ventricular systolic pressure; RVS’, right ventricular systolic excursion velocity; cm/s, centimetre per second; E/E′ wave, A A′ wave; RV, right ventricular; IVC, inferior vena cava.

We used TAPSE as it provided the highest estimate of RVD from the conventional tools. The relative risk of detecting RVD using strain analysis was 2.08 (95% CI 1.61−2.68) fold greater than in the use of TAPSE.

Over half (29/48, 60%) of the COVID-19 group had an admission P/F ratio <150 compared to 58% (14/24) of the non-COVID-19 group, *P* = 0.87. When using a P/F ratio threshold of 150, the average RVFWS did not differ between the groups.

### Mortality

The actual hospital mortality was 22.2%, which was higher than the predicted mortality rate of 4.2% (95% CI = 1.4–7%) based on the SAPS II score for the entire cohort [21 (IQR14–34)]. Despite a lower SAPS II score [18 (13–31.5)] and predicted mortality (2.9%, CI 0.5–5.3), the COVID-19 group had a significantly higher actual mortality (13/48, 27.1%) than the non-COVID-19 group (3/24, 12.5%). Significant factors on echocardiography associated with mortality were a larger RV, as evidenced by longer RV length, median of 66.5 mm in non-survivors compared to 59.5 mm in survivors (*P* = 0.04). Regarding the COVID-19 group only, higher troponin (*P* = 0.04), higher SAPS 2 (*P* = 0.02) SOFA score (*P* = 0.02), and lower pH (*P* = 0.01) were found in the non-survivors compared to the survivors. There were no significantly different right heart echocardiography findings between the COVID-19 survivors and non-survivors. See *Table 4* for factors associated with mortality.

**Table 4:** Factors associated with mortality

Variables	All survivors {*n* 56}	All non-survivors {*n* 16}	*P* value	COVID-19 survivors {35}	COVID-19 non-survivors {13}	*P* value
Time from isovolumetric contraction to the peak of the S wave (TTP) (ms)	93 [83–117] {55}	101 [90–114.5] {16}	0.85	96.5 [88–112] {34}	99.0 [90.0–112.0] {13}	0.99
TAPSE (mm)	17.6 [15.6–21.9] {56}	16.6 [14.15–21.15] {16}	0.23	17.4 [15.3–22] {35}	15.6 [14.0–21.0] {13}	0.14
RVSP (mmHg)	10 [7–23] {42}	10 [10–24] {11}	0.42	10 [10–20.3] {23}	10.0 [10.0–14.0] {11}	0.77
RVS’ (cm/s)	12.0 [10.2–13.4] {56}	12.3 [9.15–17.05] {16}	0.55	12.0 [9.6–13.8] {35}	12.7 [10.4–16.9] {13}	0.37
E (m/s)	53.92 (±16.78) {56}	55.05 (±15.67) {16}	0.81	52.3 (±15.9) {35}	56.0 (±16.2) {13}	0.55
A (m/s)	58.87 (±17.73) {55}	68.69 (±21.04) {16}	0.35	61.1 (±17.2) {35}	71.8 (±18.1) {13}	0.53
E/A	0.85 [0.75–1.16] {55}	0.82 [0.61–1.09] {16}	0.23	0.84 [0.72–1.07] {35}	0.80 [0.6–0.88] {13}	0.37
E′	10.53 (±3.00) {55}	10.02 (±2.89) {16}	0.93	9.8 (±2.3) {34}	9.5 (±2.5) {12}	0.53
A′	13.8 [11.3–15.8] {56}	15.1 [12.6–199] {15}	0.21	13.4 [11.3–15.7] {35}	15.1 [12.7–18.2] {12}	0.29
E/E′	5.30 [3.83–6.58] {55}	5.47 [4.06–7.91] {16}	0.46	5.4 [4.0–6.6] {34}	5.7 [5.0–8.1] {12}	0.33
RVFWS (%)	−13.48 (±7.08) {52}	−16.31 (±5.88) {16}	0.43	−13.5 (±7.5) {31}	−15.6 (±6.2) {13}	0.25
RV wall thickness (cm)	0.60 [0.57–0.75] {53}	0.6 [0.51–0.68] {16}	0.36	0.6 [0.54–0.69] {32}	0.6 [0.55–0.63] {13}	0.78
RV base (mm)	31.4 [27.4–36.8] {53}	30.2 [26.96–35.9] {16}	0.77	31.6 [27.4–39.3] {31}	28.2 [26.2–35.6] {13}	0.39
RV length (mm)	59.5 [51.7–66.6] {54}	66.5 [57.5–74.35] {16}	0.04^a^	58.0 [41.1–64.7] {31}	61.0 [56.8–68.5] {13}	0.14
Tricuspid annular size (mm)	29.52 (±4.90) {54}	27.11 (±5.16) {16}	0.74	29.4 (±4.6) {34}	26.2 (±5.0) {13}	0.18
IVC diameter (mm)	11.4 [9.00–13.80] {53}	10.2 [8.5–13.85] {16}	0.76	10.3 [8.8–12.1] {32}	10.2 [8.0–13.1] {13}	0.78
RA length (mm)	42.01 (±7.54) {54}	25.54 (±5.36) {16}	0.16	43.0 (±7.5) {33}	40.4 (±5.9) {13}	0.19
RA width (mm)	35.73 (±7.22) {53}	40.96 (±5.41) {16}	0.92	35.2 (±6.6) {33}	32.4 (±6.3) {13}	0.19
CRP mg/L	122 [58–193.5] {56}	137.5 [79.5–244] {16}	0.52	130 [51–180] {35}	135 [80–264] {13}	0.65
d-dimer mg/L	1.15 [0.38–2.76] {56}	1.58 [0.58–5.19] {16}	0.34	0.49 [0.35–1.73] {35}	1.01 [0.39–4.65] {13}	0.20
troponin ng/L	7 [5–17] {56}	13 [7–29] {16}	0.04^a^	6 [5–17] {35}	13 [8–25] {13}	0.04^a^

^a^Statistically significant, [IQR] interquartile range with median, (SD) standard deviation with mean, interquartile range, {} number, ms, milliseconds; mm, millimetres; mmHg, millimetre mercury; *n*, number; TAPSE, Tricuspid Annular Plane Systolic Excursion; RVSP, right ventricular systolic pressure; RVS’, right ventricular systolic excursion velocity; cm/s, centimetre per second; RVFWS, right ventricular free wall strain; RA, right atrial; E/E′, wave, A A′ wave, RV, right ventricular, IVC, inferior vena cava, LVEF, left ventricular ejection fraction, CRP, C reactive protein.

We generated a logistic regression model using six variables including an oxygenation marker (PaO_2_), a marker of ventilation (PaCO_2_), a metabolic indicator (bicarbonate), severity of illness score (SAPS II), COVID-19 status, and a marker of RV strain (average strain) to determine independent predictors of mortality. Four parameters with a *P*-value <0.5 on univariate analysis were included in the final model. A higher SAPS II score (*P* = 0.04) and COVID-19 positivity (0.05) were independent predictors of mortality.

## Discussion

Our main finding was a high prevalence (around 80% vs. 35%) of RVD amongst patients presenting with hypoxic pneumonia. RVFWS was superior to traditional RV parameters (TAPSE, RVS’, and PASP) for assessing early RV systolic dysfunction in both patient cohorts, detecting more cases of RV systolic dysfunction in both COVID-19 and non-COVID-19 pneumonia groups.

It is difficult to ascertain the true degree of RVD that occurs specifically due to COVID-19 hypoxic pneumonia owing to the confounding effects of disease severity, varying P/F ratios, clinical settings (hospitalized vs. intensive care), and variability in the timing of echocardiography with regard disease progression, however, the prevalence of RVFWS is reported to vary between 12.6%^31,32^ and 86%.^26^

Sun *et al.*^31^ demonstrated similar prevalences of RVD among COVID-19 pneumonia and bacterial pneumonia. Their prevalences were lower than ours and may be explained by the use of traditional parameters of TAPSE and RVS’. Other differences included an older population, and a very low human immunodeficiency virus (HIV) rate. In addition, neither severity of illness scores nor P/F ratios were described. *Bleakley et al*. also found a higher proportion of RVD in critically ill COVID-19 patients when using RVFWS compared to TAPSE and RVS’. Gibson *et al*.^32^ described the prevalence of abnormal RV strain in 66% of 32 mechanically ventilated COVID-19 patients. They did not find a correlation between RV strain and severity of disease or patient outcome. This suggests that RVD may not be solely attributable to alveolar collapse or be a consequence of positive pressure ventilation.

We propose that the observed similar prevalence of RVD in both COVID-19 and non-COVID-19 pneumonia is in part due to the early occurrence of hypoxia in the COVID-19 disease course, compared to the later presentation of hypoxia which is seen in non-COVID-19 pneumonia, by which time the patients have multi-organ involvement and higher disease severity scores.

RV wall thickness was greater in the non-COVID-19 than the COVID-19-pneumonia group. Wall thickness is a feature of hypertrophy, which is a compensatory adaptive response, developing over time with exposure to various stressors (high pressures and/or volumes),^33^ and its presence may suggest an element of chronicity. This suggests that the non-COVID-19 cohort may have had a component of pre-existing RVD. Since the prevalence of RVD is similar in both non-COVID-19 and COVID-19 groups, this may be suggestive of one or more additional, and possibly some acute factors (e.g. endothelial dysfunction, thrombotic state, direct myocardial effect) resulting in a similar RVD prevalence in the COVID-19 group.

We found a weak correlation between elevated troponin and impaired RV diastolic relaxation, implying subendocardial ischaemia. The possible causes include hypoxia, stress, shunting, and lung atelectasis. Furthermore, myocarditis, micro- and macro-vascular dysfunction, the cytokine storm, and the development of pulmonary emboli, all contribute to myocardial dysfunction and troponin leakage^34^ within COVID-19. There is little data on RV diastolic dysfunction with which to compare the findings of the current study. The current study did not find troponin to be a discriminating factor between COVID-19 and non-COVID-19 pneumonia. Some studies describe non-COVID-19 lung injury as having higher troponin than COVID-19-related lung injury,^35^ and others attribute the myocardial injury in COVID-19 to traditional risk factors (age and co-morbidities), resulting in less myocardial injury within the COVID-19 compared to non-COVID-19 ARDS after adjustments for risk factors.^36^

Troponin was significantly higher in all-cause non-survivors and COVID-19 non-survivors, and C reactive protein (CRP) and d-dimers trended higher, suggesting a prominent role of myocardial injury within the non-surviving group, in keeping with international literature associating troponin leakage to poor prognosis.^15^ Data from early in the COVID-19 pandemic described an elevated troponin as an independent predictor for mortality,^37^ and this has been reported in multiple studies during the later periods of the pandemic.^38–40^ *Sun et al.* demonstrated an increased accuracy for predicting death when combining highly sensitive troponin levels with RVFWS, as compared to RVFWS alone.^41^ Myocardial injury has a relationship with coagulopathy and both are associated with poor outcomes.^36^ Although d-dimer levels are raised in both COVID-19 and non-COVID-19 pneumonia, and as such are not a discriminatory test,^35^ metanalysis has shown d-dimers to predict the severity of disease and mortality in COVID-19.^42^

COVID-19 was found to be an independent predictor of mortality. Mortality was higher than predicted for COVID-19 but not for non-COVID-19 pneumonia, despite the two groups being similar in terms of oxygenation, LV function, and prevalence of RVD, and the COVID-19 group having a clinically significantly lower SAPS II score. Increased severity of illness was the only independent predictor of mortality in this study, despite other studies describing other factors, including various RV parameters.^4^ The actual mortality for the non-COVID-19 group was 12%, well within the 15% upper limit of predicted mortality for this group, compared to 27% in the COVID-19 group. Since both COVID-19 positive and negative groups were initially managed in the same unit it is unlikely that neglect due to the COVID-19 pandemic was a major contributor to poorer COVID-19 outcomes. We postulate that COVID-19 progresses from admission (when our measurements were taken), from predominantly respiratory and hypoxic pneumonia to a multi-system thrombo-inflammatory disease, causing multi-organ dysfunction, contributing to the high mortality seen despite the lower initial predicted mortality.

### Limitations

This study was conducted at a single centre, with a small sample size, and is not necessarily generalizable. Echocardiography is operator-dependent, however, this study aimed to mitigate this by having the cardiologist blinded to the clinical details and COVID-19 status of the patient. Furthermore, we used average free wall strain, compared to other investigators who used global longitudinal strain, and this may contribute to different values of strain. We excluded patients with LV systolic dysfunction but not diastolic dysfunction, which in turn was not systematically studied in all these patients. We did not conduct a propensity score-matched analysis to account for imbalances in the following variables: age, HIV, hypertension, and diabetes due to sample size, but this could potentially confound the findings, and findings should be regarded with circumspection. The impact of BMI on RVD was not assessed. Normal controls were not included in this study.

## Conclusions

Strain analysis was found to be superior to both M-mode and Doppler for detection of RVD. Despite similar oxygenation deficits in both COVID-19 and non-COVID-19 patients, the prevalence of RVD was not different. The associations between troponin, coagulopathy, and RVD are complex and future consideration should be given to the potential role of endothelialitis in the disease process.

## Data Availability

Anonymized supporting data will be made available upon request and is available in an Excel spreadsheet.

## Lead author biography

Sarah Alexandra van Blydenstein (preferred name Alex) is an adult pulmonologist from Johannesburg. She works in the Adult Pulmonology division at Chris Hani Baragwanath Academic Hospital. She is pursuing a PhD investigating COVID-19 and endothelialitis. She is a member of the sats peripheral oxygen saturation council, and Chair of the MMed Committee, University of Witwatersrand. Her interests include COVID-19, oxygen delivery systems and ventilatory support, lung cancer, sarcoidosis, preventive therapy, and developing research.

## References

[1] Paternoster G, Bertini P, Innelli P, Trambaiolo P, Landoni G, Franchi F et al Right ventricular dysfunction in patients with COVID-19: a systematic review and meta-analysis. J Cardiothorac Vasc Anesth 2021;35:3319–24. 33980426 10.1053/j.jvca.2021.04.008 PMC8038863

[2] Wu C, Chen X, Cai Y, Xia J, Zhou X, Xu S et al Risk factors associated with acute respiratory distress syndrome and death in patients with coronavirus disease 2019 pneumonia in Wuhan, China. JAMA Intern Med 2020;180:934–43. 32167524 10.1001/jamainternmed.2020.0994 PMC7070509

[3] García-Cruz E, Manzur-Sandoval D, Rascón-Sabido R, Gopar-Nieto R, Barajas-Campos RL, Jordán-Ríos A et al Critical care ultrasonography during COVID-19 pandemic: the ORACLE protocol. Echocardiography 2020;37:1353–61. 32862474 10.1111/echo.14837

[4] Mahmoud-Elsayed HM, Moody WE, Bradlow WM, Khan-Kheil AM, Senior J, Hudsmith LE et al Echocardiographic findings in patients with COVID-19 pneumonia. Can J Cardiol 2020;36:1203–7. 32474111 10.1016/j.cjca.2020.05.030 PMC7255734

[5] Corica B, Marra AM, Basili S, Cangemi R, Cittadini A, Proietti M et al Prevalence of right ventricular dysfunction and impact on all-cause death in hospitalized patients with COVID-19: a systematic review and meta-analysis. Sci Rep 2021;11:17774. 34493763 10.1038/s41598-021-96955-8 PMC8423751

[6] Shafiabadi Hassani N, Shojaee A, Khodaprast Z, Sepahvandi R, Shahrestanaki E, Rastad H. Echocardiographic features of cardiac injury related to COVID-19 and their prognostic value: a systematic review. J Intensive Care Med 2021;36:500–8. 33349095 10.1177/0885066620981015

[7] Tang N, Bai H, Chen X, Gong J, Li D, Sun Z. Anticoagulant treatment is associated with decreased mortality in severe coronavirus disease 2019 patients with coagulopathy. J Thromb Haemost 2020;18:1094–9. 32220112 10.1111/jth.14817 PMC9906401

[8] Middeldorp S, Coppens M, van Haaps TF, Foppen M, Vlaar AP, Müller MCA et al Incidence of venous thromboembolism in hospitalized patients with COVID-19. J Thromb Haemost 2020;191:145–7. 10.1111/jth.14888 PMC7497052 32369666

[9] Llitjos JF, Leclerc M, Chochois C, Monsallier JM, Ramakers M, Auvray M et al High incidence of venous thromboembolic events in anticoagulated severe COVID-19 patients. J Thromb Haemost 2020;18:1743–6. 32320517 10.1111/jth.14869 PMC7264774

[10] Wichmann D, Sperhake JP, Lütgehetmann M, Steurer S, Edler C, Heinemann A et al Autopsy findings and venous thromboembolism in patients with COVID-19: a prospective cohort study. Ann Intern Med 2020;173:268–77. 32374815 10.7326/M20-2003 PMC7240772

[11] Lodigiani C, Iapichino G, Carenzo L, Cecconi M, Ferrazzi P, Sebastian T et al Venous and arterial thromboembolic complications in COVID-19 patients admitted to an academic hospital in Milan, Italy. Thromb Res 2020;191:9–14. 32353746 10.1016/j.thromres.2020.04.024 PMC7177070

[12] Sreter KB, Budimir I, Golub A, Dorosulić Z, Sabol Pušić M, Boban M. Changes in pulmonary artery systolic pressure correlate with radiographic severity and peripheral oxygenation in adults with community-acquired pneumonia. J Clin Ultrasound 2018;46:41–7. 28940421 10.1002/jcu.22523

[13] Pagnesi M, Baldetti L, Beneduce A, Calvo F, Gramegna M, Pazzanese V et al Pulmonary hypertension and right ventricular involvement in hospitalised patients with COVID-19. Heart 2020;106:1324–31. 32675217 10.1136/heartjnl-2020-317355 PMC7476272

[14] Gattinoni L, Coppola S, Cressoni M, Busana M, Rossi S, Chiumello D. COVID-19 Does not lead to a ‘typical’ acute respiratory distress syndrome. Am J Respir Crit Care Med 2020;201:1299–300. 32228035 10.1164/rccm.202003-0817LE PMC7233352

[15] Deng Q, Hu B, Zhang Y, Wang H, Zhou X, Hu W et al Suspected myocardial injury in patients with COVID-19: evidence from front-line clinical observation in Wuhan, China. Int J Cardiol 2020;311:116–21. 32291207 10.1016/j.ijcard.2020.03.087 PMC7141178

[16] Li Y, Li H, Zhu S, Xie Y, Wang B, He L et al Prognostic value of right ventricular longitudinal strain in patients with COVID-19. JACC Cardiovasc Imaging 2020;13:2287–99. 32654963 10.1016/j.jcmg.2020.04.014 PMC7195441

[17] Agasthi P, Chao CJ, Siegel RJ, Pujari SH, Mookadam F, Venepally NR et al Comparison of echocardiographic parameters with cardiac magnetic resonance imaging in the assessment of right ventricular function. Echocardiography 2020;37:1792–802. 33012034 10.1111/echo.14877

[18] Coronavirus Disease 2019 (COVID-19) Treatment Guidelines:456. 34003615

[19] Le Gall JR, Lemeshow S, Saulnier F. A new simplified acute physiology score (SAPS II) based on a European/North American Multicenter Study. JAMA 1993;270:2957–63. 8254858 10.1001/jama.270.24.2957

[20] Vincent JL, de Mendonça A, Cantraine F, Moreno R, Takala J, Suter PM et al Use of the SOFA score to assess the incidence of organ dysfunction/failure in intensive care units: results of a multicenter, prospective study. Working group on ‘sepsis-related problems’ of the European Society of Intensive Care Medicine. Crit Care Med 1998;26:1793–800. 9824069 10.1097/00003246-199811000-00016

[21] Parsaee M, Ghaderi F, Alizadehasl A, Bakhshandeh H. Time from the beginning of the right ventricle isovolumetric contraction to the peak of the S wave: a new TDI indicator for the non-invasive estimation of pulmonary hypertension. Res Cardiovasc Med 2016;5:e26494. 27800451 10.5812/cardiovascmed.26494 PMC5075392

[22] Rudski LG, Lai WW, Afilalo J, Hua L, Handschumacher MD, Chandrasekaran K et al Guidelines for the echocardiographic assessment of the right heart in adults: a report from the American Society of Echocardiography endorsed by the European Association of Echocardiography, a Registered Branch of the European Society of Cardiology, and the Canadian Society of Echocardiography. J Am Soc Echocardiogr. 2010;23:685–713; quiz 786–8. 20620859 10.1016/j.echo.2010.05.010

[23] Nel S, Nihoyannopoulos P, Libhaber E, Essop MR, Ferreira Dos Santos C, Matioda H et al Echocardiographic indices of the left and right heart in a normal black African population. J Am Soc Echocardiogr 2020;33:358–67. 31959528 10.1016/j.echo.2019.10.009

[24] Meel R, Peters F, Libhaber E, Essop ME. Unmasking right ventricular dysfunction in chronic rheumatic mitral regurgitation. Cardiovasc J Afr 2019;30:216–21. 31140546 10.5830/CVJA-2019-020

[25] La Gerche A, Jurcut R, Voigt JU. Right ventricular function by strain echocardiography. Curr Opin Cardiol 2010;25:430–6. 20592586 10.1097/HCO.0b013e32833b5f94

[26] Bleakley C, Singh S, Garfield B, Morosin M, Surkova E, Mandalia MS et al Right ventricular dysfunction in critically ill COVID-19 ARDS. Int J Cardiol 2021;327:251–8. 33242508 10.1016/j.ijcard.2020.11.043 PMC7681038

[27] Longobardo L, Suma V, Jain R, Carerj S, Zito C, Zwicke DL et al Role of two-dimensional speckle-tracking echocardiography strain in the assessment of right ventricular systolic function and comparison with conventional parameters. J Am Soc Echocardiogr 2017;30:937–946.e6. 28803684 10.1016/j.echo.2017.06.016

[28] Morris DA, Krisper M, Nakatani S, Köhncke C, Otsuji Y, Belyavskiy E et al Normal range and usefulness of right ventricular systolic strain to detect subtle right ventricular systolic abnormalities in patients with heart failure: a multicentre study. Eur Heart J Cardiovasc Imaging 2017;18:212–23. 26873461 10.1093/ehjci/jew011

[29] Harris PA, Taylor R, Thielke R, Payne J, Gonzalez N, Conde JG. Research electronic data capture (REDCap)–a metadata-driven methodology and workflow process for providing translational research informatics support. J Biomed Inform 2009;42:377–81. 18929686 10.1016/j.jbi.2008.08.010 PMC2700030

[30] Harris PA, Taylor R, Minor BL, Elliott V, Fernandez M, O’Neal L et al The REDCap consortium: building an international community of software platform partners. J Biomed Inform 2019;95:103208. 31078660 10.1016/j.jbi.2019.103208 PMC7254481

[31] Sun K, Cedarbaum E, Hill CA, Win S, Parikh NI, Hsue PY et al Association of right ventricular dilation on echocardiogram with in-hospital mortality among patients hospitalized with COVID-19 compared with bacterial pneumonia. J Am Soc Echocardiogr 2023;36:558–62. 36592874 10.1016/j.echo.2022.12.019 PMC9803370

[32] Gibson LE, Fenza RD, Lang M, Capriles MI, Li MD, Kalpathy-Cramer J et al Right ventricular strain is common in intubated COVID-19 patients and does not reflect severity of respiratory illness. J Intensive Care Med 2021;36:900–9. 33783269 10.1177/08850666211006335 PMC8267080

[33] Frey N, Katus HA, Olson EN, Hill JA. Hypertrophy of the heart. Circulation 2004;109:1580–9. 15066961 10.1161/01.CIR.0000120390.68287.BB

[34] Park JF, Banerjee S, Umar S. In the eye of the storm: the right ventricle in COVID-19. Pulm Circ 2020;10:2045894020936660. 10.1177/2045894020936660 PMC7333504 32655856

[35] Zhang J, Huang X, Ding D, Zhang J, Xu L, Hu Z et al Comparative study of acute lung injury in COVID-19 and non-COVID-19 patients. Front Med 2021;8:666629. 10.3389/fmed.2021.666629 PMC8415545 34485324

[36] Metkus TS, Sokoll LJ, Barth AS, Czarny MJ, Hays AG, Lowenstein CJ et al Myocardial injury in severe COVID-19 compared with non-COVID-19 acute respiratory distress syndrome. Circulation 2021;143:553–65. 33186055 10.1161/CIRCULATIONAHA.120.050543 PMC7864609

[37] Nie SF, Yu M, Xie T, Yang F, Wang HB, Wang ZH et al Cardiac troponin I is an independent predictor for mortality in hospitalized patients with COVID-19. Circulation 2020;142:608–10. 32539541 10.1161/CIRCULATIONAHA.120.048789 PMC7418761

[38] Salvatici M, Barbieri B, Cioffi SMG, Morenghi E, Leone FP, Maura F et al Association between cardiac troponin I and mortality in patients with COVID-19. Biomarkers 2020;25:634–40. 33003961 10.1080/1354750X.2020.1831609 PMC7711728

[39] García de Guadiana-Romualdo L, Morell-García D, Rodríguez-Fraga O, Morales-Indiano C, María Lourdes Padilla Jiménez A, Gutiérrez Revilla JI et al Cardiac troponin and COVID-19 severity: results from BIOCOVID study. Eur J Clin Invest 2021;51:e13532. 33660278 10.1111/eci.13532 PMC7995181

[40] Javidi Dasht Bayaz R, Askari VR, Tayyebi M, Ahmadi M, Heidari-Bakavoli A, Baradaran Rahimi V. Increasing cardiac troponin-I level as a cardiac injury index correlates with in-hospital mortality and biofactors in severe hospitalised COVID-19 patients. J Infect Chemother 2023;29:250–6. 36414196 10.1016/j.jiac.2022.11.007 PMC9674565

[41] Sun W, Zhang Y, Wu C, Xie Y, Peng L, Nie X et al Incremental prognostic value of biventricular longitudinal strain and high-sensitivity troponin I in COVID-19 patients. Echocardiography 2021;38:1272–81. 34184314 10.1111/echo.15133 PMC8444873

[42] Sakka M, Connors JM, Hékimian G, Martin-Toutain I, Crichi B, Colmegna I et al Association between D-dimer levels and mortality in patients with coronavirus disease 2019 (COVID-19): a systematic review and pooled analysis. J Med Vasc 2020;45:268–74. 32862984 10.1016/j.jdmv.2020.05.003 PMC7250752

